# Global Lysine Acetylome Analysis of LPS-Stimulated HepG2 Cells Identified Hyperacetylation of PKM2 as a Metabolic Regulator in Sepsis

**DOI:** 10.3390/ijms22168529

**Published:** 2021-08-08

**Authors:** Ann-Yae Na, Sanjita Paudel, Soyoung Choi, Jun Hyung Lee, Min-Sik Kim, Jong-Sup Bae, Sangkyu Lee

**Affiliations:** 1 BK21 FOUR Community-Based Intelligent Novel Drug Discovery Education Unit, College of Pharmacy and Research Institute of Pharmaceutical Sciences, Kyungpook National University, Daegu 41566, Korea; cpblady@daum.net (A.-Y.N.); sanjitapdl99@gmail.com (S.P.); sylvdh@naver.com (S.C.); baejs@knu.ac.kr (J.-S.B.); 2 Department of New Biology, Daegu Gyeongbuk Institute of Science & Technology, Daegu 42988, Korea; jhlee8810@dgist.ac.kr (J.H.L.); mkim@dgist.ac.kr (M.-S.K.)

**Keywords:** sepsis-induced liver dysfunction, hyperacetylation, SIRT, lysine acetylation, pyruvate kinase M2

## Abstract

Sepsis-induced liver dysfunction (SILD) is a common event and is strongly associated with mortality. Establishing a causative link between protein post-translational modification and diseases is challenging. We studied the relationship among lysine acetylation (Kac), sirtuin (SIRTs), and the factors involved in SILD, which was induced in LPS-stimulated HepG2 cells. Protein hyperacetylation was observed according to SIRTs reduction after LPS treatment for 24 h. We identified 1449 Kac sites based on comparative acetylome analysis and quantified 1086 Kac sites on 410 proteins for acetylation. Interestingly, the upregulated Kac proteins are enriched in glycolysis/gluconeogenesis pathways in the Kyoto Encyclopedia of Genes and Genomes (KEGG) category. Among the proteins in the glycolysis pathway, hyperacetylation, a key regulator of lactate level in sepsis, was observed at three pyruvate kinase M2 (PKM2) sites. Hyperacetylation of PKM2 induced an increase in its activity, consequently increasing the lactate concentration. In conclusion, this study is the first to conduct global profiling of Kac, suggesting that the Kac mechanism of PKM2 in glycolysis is associated with sepsis. Moreover, it helps to further understand the systematic information regarding hyperacetylation during the sepsis process.

## 1. Introduction

Sepsis, a life-threatening complication, occurs when the body’s response to infection causes injuries to tissues and organs. During the initial stage of the disease, almost all patients with sepsis exhibit excessive hyperinflammation characterized by a cytokine storm, overproduction of reactive oxygen species, and metabolic shift [1]. Inflammatory cytokines include tumor necrosis factor-α (TNF-α) and interleukin-1β (IL-1β) [2] as well as high mobility group box 1 (HMGB1) [3,4]. Liver dysfunction during sepsis is one of the components of multiple organ dysfunction syndrome and is usually associated with a poor prognosis. However, the exact mechanism remains unclear. Although the liver plays a pivotal role in regulating a wide range of metabolic, homogeneous, and host defense activities, liver dysfunction is generally considered a result of shock and early tissue hypoperfusion [5]. Moreover, sepsis can cause impaired liver function, contributing to the high mortality associated with sepsis. Liver dysfunction represents a specific and independent risk factor for poor prognosis in critically ill patients [6], prone to developing sepsis, sepsis-associated organ failure, and death owing to pre-existing liver disease [7]. Unfortunately, no specific therapeutics for SILD are available at present [8].

Protein post-translational modification of lysine residues influences protein structure and function and plays a critical role in regulating nearly every aspect of cellular biology [9,10]. Although lysine acetylation (Kac) is considered essential for nuclear modifications in histone [11], it was recently shown that the regulatory implications of the modification extend beyond gene regulation. Changes in cellular Kac status can induce changes in metabolic enzyme activity and provide a cellular mechanism to adapt to metabolic changes [12,13]. In addition, Kac is highly reversible, wherein a lysine residue is acetylated by the action of the histone/lysine acetyltransferase enzymes (HATs/KATs) and is removed by histone deacetylases (HDACs) and sirtuins (SIRTs).

The peptides from the SIRTs are associated with critical metabolic proteins and regulate various metabolic processes [14,15], including insulin secretion, cell cycle, and apoptosis [16]. Seven isoforms of the SIRT family have been identified in different localization in humans—SIRT1, SIRT6, and SIRT7 are predominantly found in the nucleus and SIRT3, SIRT4, and SIRT5 are mitochondrial [17]. SIRTs are associated with inflammation metabolism during sepsis. A report on the role of SIRT in inflammation showed that NAD^+^ levels as well as SIRT transcription and protein levels continue to decrease in specific tissues during acute inflammation [17]. Among the functional roles of SIRT in inflammation, deacetylation activity by SIRT1 is most likely affected; however, ADP ribosylation (SIRT4) and removal of succinyl, malonyl, and glutamyl groups from lysine residues (SIRT5) may be associated with inflammation [18,19].

Although it was reported that the SIRTs level was reduced in the inflammatory response in sepsis [18,20], leading to hyperacetylation in the cellular process, there is no systematic information on lysine acetylome in SILD. Therefore, more studies are warranted to understand the factors regulating novel pathways, based on the global acetylome profiling linked with the progression of SILD, and identify the substrates of SIRTs. Here, we investigated the impact of protein hyperacetylation in SILD that could emerge as a critical regulator through SIRTs. Our study aimed to present data on global profiling of sepsis-related protein interaction network, biological processes, KEGG pathway, and related enzymes responsible for protein acetylation.

## 2. Results

### 2.1. Endotoxin-Induced Inflammation Response in Human Hepatocytes

**Figure 1:**
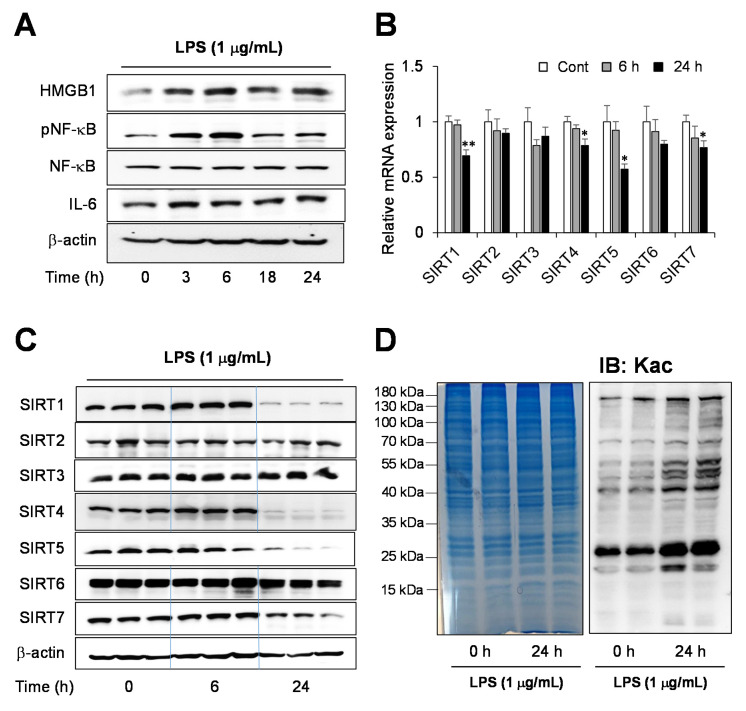
SIRTs-mediated lysine acetylation (Kac) levels in sepsis-induced liver dysfunction (SILD). (**A**) The level of inflammatory factors determined using Western blotting. HepG2 cells were incubated with LPS (1 μg/mL) for different durations. (**B**) mRNA expression of SIRT 1-7 in LPS-treated HepG2 cells. The expression ratio represents the change in the ratio relative to β-actin compared with the untreated group (0 h). Band intensities were quantified from three independent experiments. (**C**) Western blot analysis of SIRT 1–7 proteins. (**D**) Increased global Kac in the LPS-treated HepG2 (1 μg/mL). The data are presented as the means ± SD of three independent experiments. Statistical significance was assessed using one-way ANOVA followed by Bonferroni post-hoc test and is represented as follows: * *p* < 0.05 and ** *p* < 0.01 vs. control.

Human hepatoma HepG2 cells were treated with LPS to establish an in vitro sepsis model and the secretion of inflammatory cytokines in sepsis was increased. We assessed changes in the protein levels of proinflammatory cytokines with Western blotting (Figure 1A and Figure S1A). The proinflammatory factor, NF-κB p65 phosphorylation level, and IL-6 protein expression level were measured in a time-dependent manner. The NF-κB phosphorylation gradually increased 3 and 6 h after the LPS treatment and decreased from 18 h onward. HMGB1, a critical inflammatory mediator, was released during sepsis, increasing 3–24 h after LPS treatment. We were interested in studying the regulation of pathological Kac in SILD. The gene and protein expression levels of the SIRTs 1–7, which play a role in the deacylase of lysine acylation (Figure 1B,C and Figure S1B), were time-dependently monitored after the LPS treatment. The mRNA and protein levels showed the same results, with the SIRTs 1, 4, 5, and 7 being reduced after LPS treatment for the 24 h group. The expressions of SIRT 2, 3, and 6 were not altered. After 24 h of treatment, the expression of SIRT1, present in the cytosol and nucleus, decreased dramatically in the LPS-treated cells. According to decreased SIRTs expression level, the protein Kac levels were altered during this process (Figure 1D). Samples with 6 and 24 h of treatment, having the highest inflammatory response after the LPS treatment, were used in the subsequent proteome analysis, whereas samples with 24 h treatment, with a significant decrease in expressed SIRTs, were used in the acetylome analysis.

### 2.2. Quantitative Proteomics for LPS-Induced Sepsis in HepG2 Cells

**Figure 2:**
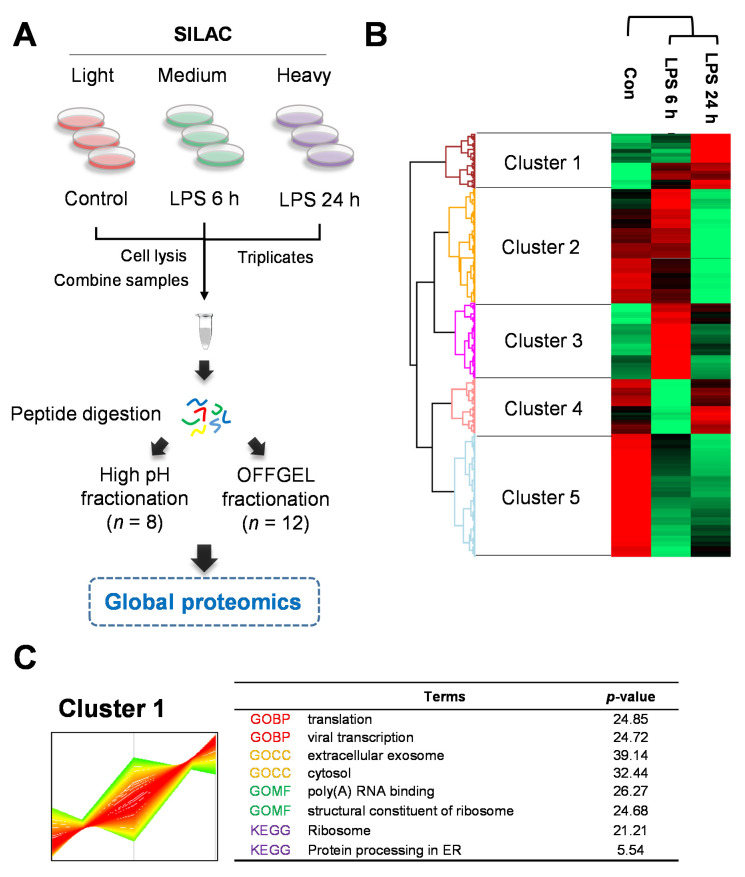
Experimental scheme of comparative proteome analysis in sepsis-induced liver dysfunction (SILD) in human hepatoma cells. (**A**) For the global proteomic approach, SILAC-labeled HepG2 cells were prepared with 1 μg/mL LPS treatment for 6 h (“medium”, K4R6) and 24 h (“heavy”, K8R10) as well as without LPS treatment as the control group (“light”, K0R0). Peptide were subjected to fractionation using two methods: high-pH phase fractionation and the OFF-GEL fraction. All experiments were performed in biological triplicates. (**B**) The global proteomics analysis of significantly differentially expressed proteins (DEPs) by volcano plot of LPS treatment for the 6 h and 24 h group. (**C**) Protein expression dynamic clusters based on global protein expression patterns at each stage of sepsis-induced liver dysfunction (SILD). The functional gene ontology (GO) and KEGG were shown at cluster 1.

A general workflow to perform the LC–MS/MS-based identification and the SILAC-based quantitation of the global proteomic is summarized in Figure 2A. The SILAC-based metabolic labeling was performed for the quantitative evaluation using HepG2 cells, which are human hepatoma cells. The HepG2 cells were grown in SILAC medium containing isotope-labeled lysine and arginine to construct SILAC cell lines named “medium” (K4R6) and “heavy” (K8R10), respectively. Each substitution rate was 98.19% and 96.06%. For relative quantification, the protein was obtained as medium-label cells for 6 h LPS-treated cells and heavy-labeled cells for 24 h LPS-treated cells. The trypsin digested peptides were subjected to two different fractionation methods, the high-pH phase fractionation and the OFF-GEL fractionation, to increase identified proteins (Figure 2A). Moreover, we performed the analysis in two kinds of high-resolution and accurate MS: the Velos-Orbitrap and the Q-Exactive hybrid quadrupole-orbitrap, respectively. The merged MS/MS spectra were analyzed using a MaxQuant 1.5, querying the Uniprot human database to identify and quantify proteins. The search data was cleaned with the removal of potential contaminants, corresponding to <1% of the false-positive proteins and those identified only by a site modification, to increase the accurate identification of proteins.

Overall, we identified 3167 proteins and quantified 2256 (71.2%) proteins in LPS-treated HepG2 cells (Figure S2A and Table S1). The differentially expressed proteins (DEPs) were 235 proteins (102 upregulated proteins, 133 downregulated) in LPS-treated cells for 6 h and 228 proteins (48 upregulated, 180 downregulated) in LPS-treated cells for 24 h, the ratio of which was changed >1.5-fold or <0.666-fold (Figure S2A). For the global proteomics analysis, we performed three independent experiments to obtain the peptide samples. Triplicate analysis revealed 1115 overlapped LPS proteins in the 24 h group in the Venn diagram (Figure S2B). Moreover, the correlation was approximately 0.645–0.742 in each experiment set (Figure S3).

Heat maps of quantitative proteins were created to identify protein clusters with similar patterns throughout the sepsis process (Figure 2B). The Z-score was used to calculate the quantitative ratio of the proteins. A red–green plot was used to improve the visualization. This analysis generated five clusters (1–5), including 293, 611, 405, 290, and 657 quantified proteins, respectively (Figure 2C and Figure S3). Proteins for each cluster are shown in Table S2. Cluster 1 showed a pattern that gradually increased to 24 h depending on the LPS treatment in HepG2 cells (Figure 2C). Based on the translation category in GOBP, ribosome categories in GOMF, and KEGG, cluster 1 contains proteins related to protein synthesis such as ribosomal proteins. Clusters 2 and 5 have different patterns at 6 h but showed a decreasing trend at 24 h (Figure S4). The carbon metabolism, spliceosome, and metabolic pathways in the KEGG category are commonly included in clusters 2 and 5, and the energy metabolism-related functions decreased with the LPS treatment. Clusters 3 and 4 are the protein groups that increased and decreased, respectively, at 6 h but eventually recovered at 24 h.

### 2.3. Quantitative Global Lysine Acetylome Profiling in LPS-Induced SILD

**Figure 3:**
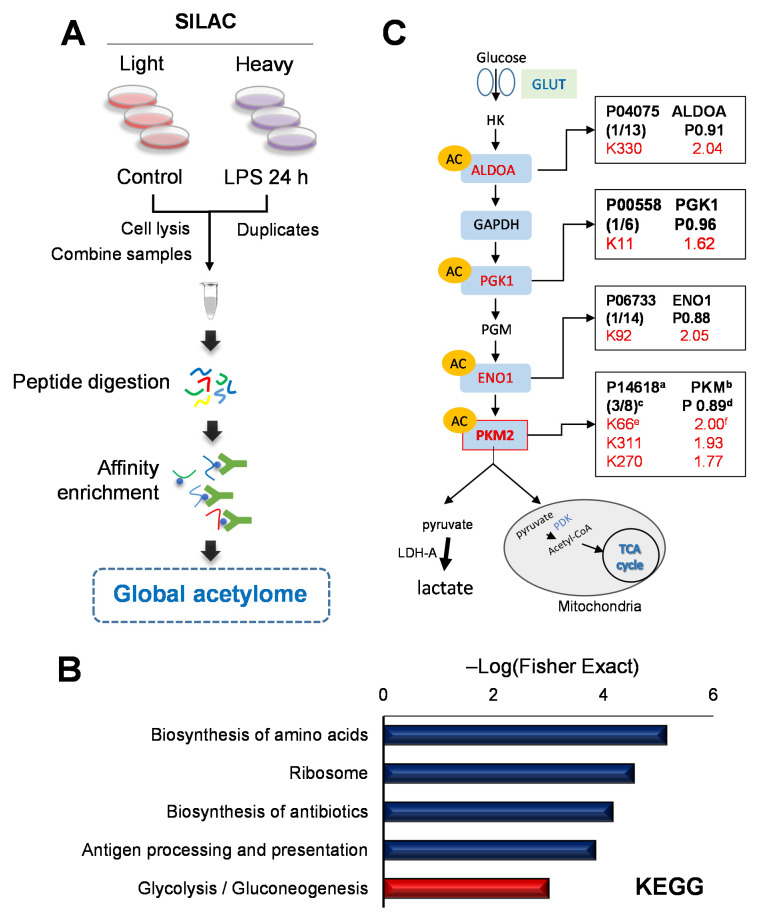
Characterization of lysine acetylation (Kac) in sepsis-induced liver dysfunction (SILD) in human hepatoma cells. (**A**) For the global lysine acetylome study, control (“light”, K0R0) and 1 μg/mL LPS treatment for the 24 h (“heavy”, K8R10) groups were prepared, and 1 mg of mixed peptides was produced. Immunoprecipitation was performed using pan-anti-Kac agarose beads. All experiments were performed in biological duplicates. (**B**) The KEGG pathway in upregulated Kac. (**C**) Upregulated Kac protein in the glycolysis pathway. ^a^, Uniprot number; ^b^, gene name; ^c^, number of upregulated Kac sites/all quantified Kac sites in protein; ^d^, quantified protein ratio (24 h vs. 0 h); ^e^, Kac position; ^f^, relative Kac intensity ratio (24 h vs. 0 h).

To identify the global acetylome, as shown in Figure 1B,C, it was confirmed that the levels of SIRTs, which are deacetylases, decreased 24 h after the LPS treatment. We collected lysates after LPS (1 μg/mL) treatment for 24 h. We developed a workflow involving a global immune affinity enrichment of the acetylated peptides coupled with identification by LC–MS/MS (Figure 3A). The Kac proteins were normalized with their protein ratio. Among all identified acetylated proteins, 1086 were quantified, including 133 DEPs (58 upregulated proteins and 75 downregulated) in the LPS-treated cells for 24 h, the ratio of which changed >1.5-fold or <0.666-fold (Table S3). To analyze the distribution of acetylation at the protein level, we calculated the number of Kac sites on each protein (Figure S5A). All experiment was analyzed with independent duplicates. The Pearson correlation coefficients of the ratio of Kac peptide intensity were 0.817 (Figure S5B). The upregulated acetylated proteins are presented in Table 1.

We performed GO and KEGG pathway analysis by calculating *p*-values of the Fisher’s exact test (Figure 3B and Figure S5C) to characterize the upregulated acetyl proteins with decreasing SIRTs. In the KEGG pathway analysis, the glycolysis and gluconeogenesis categories were enriched with canonical glycolysis in the GOMF category. The proteins interaction network showed a strong interaction with four proteins among those associated with glycolysis pathway (Figure S6), including aldolase (ALDOA), phosphoglycerate kinase 1 (PGK1), enolase1 (ENO1), and PKM2. We observed that the metabolic enzyme acetylation, a well-known process from several previous studies [21,22], increased in the glycolysis pathway. We confirmed that Kac increased at three sites in the PKM (Figure 3C).

### 2.4. Functional Analysis of the Acetylation Site of PKM2 on Glycolysis

**Figure 4:**
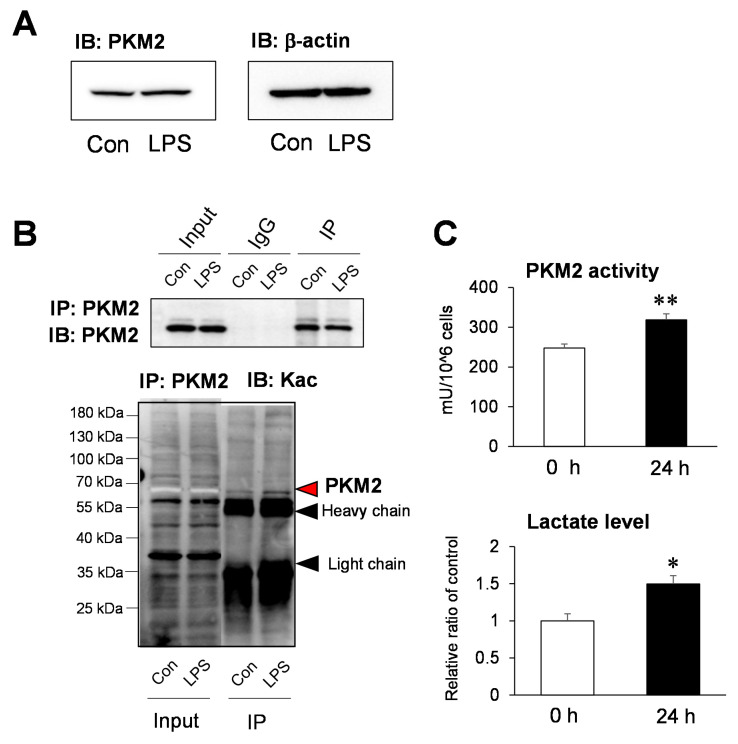
Pyruvate kinase M2 (PKM2) acetylation levels in sepsis-induced liver dysfunction (SILD). (**A**) Validation of comparative proteome analysis using Western blotting. Western blot analysis for PKM2 protein expression. (**B**) The PKM and PKM acetylation levels were determined using Western blotting after immunoprecipitation using an anti-PKM antibody. (**C**) Pyruvate kinase (PK) activity determination of control and LPS-treated HepG2 cells for 24 h. The PK activity was measured using ELISA (570 nm) (*n* = 5). The comparison of the lactate level in control and LPS-treated HepG2 cells for 24 h (*n* = 3). Data are mean ± SE. Statistical significance was assessed using student’s *t*-test and is represented as follows: * *p* < 0.05 and ** *p* < 0.01 vs. control.

It was observed that the amount of PKM2 protein did not change in the immunoblot analysis of LPS-stimulated HepG2 cells after 24 h, which was consistent with the result of global proteome analysis (Figure 4A and Figure S7A). To verify the Kac change of PKM2 in global acetylome data, after selective isolation of PKM2 protein using an anti-PKM2 antibody, immunoblotting was performed using a pan-Kac antibody. It was confirmed that acetylation increased in PKM2 after the LPS treatment (Figure 4B and Figure S7B). Moreover, we observed that PKM2 activity and lactate significantly increased in LPS-stimulated HepG2 cells (Figure 4C). If we comprehensively review the aforementioned results, the SIRTs decreased, resulting in the hyperacetylation of intracellular proteins with the LPS-induced SILD in hepatocytes. The PKM2 hyperacetylation increases the PKM2 activity and intracellular lactate concentration, which may be closely related to hepatotoxicity.

## 3. Discussion

There is consistently reliable evidence that SIRTs can control the inflammatory response induced in sepsis [16,23,24]. In the SIRT family, the role of SIRT1 in sepsis was reported in the most diverse manner, wherein the SIRT1 activation inhibits the inflammatory response caused by sepsis. When SIRT1 is activated in cecum ligation and puncture (CLP) models and LPS-induced cell lines, the expression, translocation, and release of HMGB1, which are the most important factors in sepsis, decreased [25,26,27]. In contrast, the inhibition of SIRT1 increased the gene expression and inflammatory response of TNF-a, whereas the excretion of cytokine decreased [28,29,30]. SIRTs were confirmed to be related to inflammatory response in many cells [20]. As SIRT1 is an NAD^+^-dependent deacetylase, when SIRT1 is reduced in LPS-induced SILD in HepG2 cells, the substrate proteins hyperacetylate, playing an essential role in the progression of inflammation. For example, SIRT1 deacetylates and deactivates NF-ĸB p65 and consequently downregulates the anti-inflammatory activity during inflammation caused by direct NF-κB p65 deacetylation and the HIF-1α and PGC-1α pathway [16]. Therefore, a comprehensive acetylome analysis might identify the novel regulatory factors involved in SILD affected by SIRT1.

Our study confirmed that the LPS-induced SILD in HepG2 cells decreased SIRT4 and SIRT5 along with SIRT1 (Figure 1C). Although the expression of SIRT4 is significantly reduced in an LPS-induced endothelial dysfunction model [18], SIRT4, unlike other sirtuins, primarily functions as a histone ADP-ribosyltransferase but lacks NAD-dependent deacetylase activity [31]. A decrease in SIRT5 was observed. Although SIRT5 has deacetylase activity, it is known that it has more robust activities as desuccinylase, demalonylase, and deglutarylase in several human diseases, such as cancer, Alzheimer’s, and Parkinson’s disease [19,32]. It is highly likely that the relationship between the increased Kac and SIRT4 as well as SIRT 5 in HepG2 cells with LPS-induced SILD is relatively weak compared with the decreased SIRT1. However, the relevance of the characteristic SIRT involved in the identified individual hyperacetylated proteins should be evaluated in further studies.

Here, we conducted a quantitative acetylome study to characterize the hyperacetylation proteins as the SIRT family decreased in SILD. Among the results, we observed characteristic hyperacetylation of proteins on the glycolysis pathway (Figure 3C). Based on the results from the KEGG analysis regarding the upregulated acetylome, the acetylation increased in four proteins—ALDOA, PGK1, ENO1, and PKM2—in the glycolysis pathways. The sepsis metabolism is associated with various metabolic changes, including insulin resistance, hyperlactatemia, hypoglutaminemia, hypercatabolism, and muscle wasting [33,34,35,36]. In particular, an increase in lactate concentration, a diagnostic marker in the early stages of sepsis, is thought to be a sign of acute organ dysfunction [37]. As the liver is responsible for 60% of the systemic lactate metabolism and vulnerable to acute circulatory dysfunction associated with sepsis, the change in lactate concentration is closely related to liver injury [38]. Many studies on lactate mechanisms have suggested that glycolysis is responsible for hyperlactatemia in sepsis [39].

Among the four proteins hyperacetylated in the glycolysis pathway, we studied the hyperacetylation of PKM2 as a critical regulator causing an increase in lactate levels. The increased conversion of glucose to lactate is a vital feature of the Warburg effect, increasing *PKM2* expression and facilitating lactate production [40]. Furthermore, PKM2 catalyzes the last step of glycolysis, a protein kinase and transcriptional co-activator, functioning as an essential aerobic glycolysis mediator in sepsis and promotes lactate formation [41]. Conversely, the expression of genes encoding enzymes involved in glycolysis and lactate metabolism and the synthesis of membrane transporters, i.e., glucose transporter (GLUT-1), hexokinase-3, PKM2, subunit A of LDH, and MCT4, are significantly increased in patients with sepsis [42]. Therefore, it is clear that changes in the PKM expression and activity are involved in changes in lactate concentration and can lead to sepsis, particularly SILD.

Interestingly, in our study, the PKM2 protein expression did not affect the LPS-induced SILD in HepG2 cells. Indeed, the PKM2 activity increased compared with hyperacetylation (Figure 4). To date, the role of lysine acylation in the PKM2 activity regulation has not been identified. For example, SIRT1 as deacetylase may regulate the PKM acetylation level and their catalytic activity in brain tissue [43]. Moreover, PKM2 acetylation at K305 decreases PKM2 activity through chaperone-mediated autophagy and subsequent lysosome degradation [44]. Furthermore, it was observed that the PKM2 hyperacetylation state increases the PKM activity; however, further studies are warranted to determine whether specific acetylation is significantly involved. In this study, PKM2 is a known physiological substrate of SIRT5 and SIRT5-regulated hypersuccinylation, inhibiting the pyruvate kinase activity in LPS-activated macrophages [45]. We did not consider the effect of hypersuccinylation because the PKM activity increased compared with that of the decreased SIRT5.

We highlighted the biological importance of crosstalk between the complex network of glycolysis pathway and hyperlactatemia at lysine acetylation. SIRT1, 4, and 5 decreased in the LPS-induced septic hepatocytes, leading to hyperacetylation of intracellular proteins. In particular, the hyperacetylation of PKM2 may be closely related to hepatotoxicity by increasing PKM2 activity and increasing intracellular lactate concentrations as a regulator of glycolysis. This study is limited by its use of an in vitro sepsis model to study an in vivo pathological situation. In further studies, the relationship between SIRT and acetylome in in vivo sepsis models, such as the CLP model, should be studied. It will be necessary to perform both in vitro and in vivo experiments to identify the Kac-related targets for the treatment of sepsis.

## 4. Materials & Methods

### 4.1. Cell Culture and Chemicals

The human liver HepG2 cell was maintained in DMEM (HyClone™, cytiva, Utah, UT, USA) supplemented with 10% fetal bovine serum (GE Healthcare, Little Chalfont, UK) and 1% penicillin (Gibco, Rockville, MD, USA) in a humidified incubator containing 5% CO_2_ at 37 °C. The cells were passaged upon reaching approximately 85% confluence in 150 mm culture dishes. LPS from an *E. coli* strain was purchased from Sigma-Aldrich (St. Louis, MO, USA), treated at the indicated concentrations, and then subsequent experiments were performed. For lactate determination, lactate, *O*-benzylhydroxylamine (*O*-BHA), and 1-ethyl-3-(3dimethylaminopropyl) carbodiimide (EDC) were procured from Sigma-Aldrich (St. Louis, MO, USA).

### 4.2. RNA Preparation and Quantitative Real-Time PCR

According to the manufacturer’s protocol, total cellular RNA was isolated using Trizol reagent (Invitrogen, Carlsbad, CA, USA). cDNA was synthesized using 1 µg of total RNA digested using the SuperScript III First-Strand kit (Invitrogen, Carlsbad, CA, USA). The obtained cDNA was amplified in real-time PCR using the Luna universal qPCR Master mix (New England Biolabs, MA, USA). The specific primers for the SIRT family are listed in Table S4A. The cycling protocol was 94 °C for 90 s, followed by 40 denaturation cycles with heating at 98 °C for 10 s, annealing at 58 °C for 25 s, and extension at 72 °C for 25 s. All the reactions were performed with technical duplicates and repeated independently thrice. Relative fold changes were calculated using the 2^−ΔΔ*Ct*^ method and normalized against housekeeping gene β-actin.

### 4.3. Western Blot

The cell lysates of HepG2 were sonicated, and the proteins were extracted in RIPA buffer, including protease inhibitor cocktail (Thermo Fisher Scientific, Waltham, MA, USA). A total of 10 μg of proteins were loaded to 10% sodium dodecyl sulfate-polyacrylamide (SDS-PAGE) gel. Following transfer onto polyvinylidene fluoride (PVDF) membranes (0.2 μm pore size, GE Healthcare, Chicago, IL, USA), 5% bovine serum albumin was used to block the membranes. Later, the blot was incubated with a specific primary antibody. Tris-buffered saline containing 0.05% Tween 20 was used to wash the membranes thrice for 15 min each time. Primary antibodies were diluted 1:1000 each for detecting target proteins. The secondary antibody (either rabbit or mouse) was incubated at room temperature for 2 h. After the three-time wash proceeds, and we detected blots using ECL Prime solution (GE Healthcare). Table S5 contains information about antibodies.

### 4.4. SILAC Label and Proteomics Sample Preparation for Global Proteomes

For the LC-MS/MS analysis, the HepG2 cells were cultured and labeled using SILAC media according to the following classes: “light,” “medium,” or “heavy.” The “light” cells had Lys and Arg labeling (K0R0), the “medium” cells had Lys^4^ (D_4_) and Arg^6^ (^13^C_6_) labeling (K4R6), and the “heavy” cells had Lys8 (^13^C_6_-^15^N_2_) and Arg10 (^13^C_6_-^15^N_4_) labeling (K8R10) isotope of lysine and arginine for at least four cell doublings to allow complete label incorporation. At 70% confluency, LPS was added to the culture, and 6 h later, the “medium” labeled cells were harvested. The “heavy” labeled cells were supplemented with LPS for 24 h, and the “light” labeled cells were cultured as control. The harvested cell pellet was collected and then lysed with RIPA buffer containing Halt protease inhibitor (Thermo Fisher Scientific) on ice. Reduction and alkylation were performed using 10 mM DTT (pH 8), incubated for 1 h at 37 °C. Then, iodoacetamide was added to a final concentration of 50 mM, and the resultant mixture was incubated for 45 min at room temperature in darkness. The mixture was incubated in trypsin (Promega sequencing grade, Madison, WI, USA; 1:25 protease to protein ratio) at 37 °C overnight, at pH 7–8. The addition of 1% TFA stopped digestion, and the sample was then dried in a speed vacuum.

### 4.5. Global Immunoprecipitation

A total of 1 mg of purified peptide was dissolved in NETN (40 mM Tris-HCl, pH 8.0; 200 mM NaCl; 2 mM EDTA, 1% NP-40) buffer. Next, 20 μL of anti-acetyllysine antibody-conjugated premium beads (PTM Biolab, Hangzhou, China) were prepared with PBS washing and incubated on a rotator at 4 °C for 18 h. The beads were washed thrice with 1 mL of NETN and twice with 1 mL of ETN (40 mM Tris-HCl, pH 8.0; 200 mM NaCl; 2 mM EDTA). The LC–MS-grade water was used for last wash and the mixture was centrifuged at 1000× *g* for 2 min at 4 °C between every wash step. The enriched Kac peptides were eluted in 150 μL of 0.15% TFA and gently mixed for 10 min. Then, 120 μL of the eluted peptide were transferred into a new tube, dried, and then desalted twice using a C18 ziptip.

### 4.6. Nano LC–MS/MS Analysis

The peptides were dissolved in solvent A (99.9% water with 0.1% formic acid). Then, they were analyzed using an LTQ Velos-Orbitrap Mass Spectrometer (Thermo Fisher Scientific) equipped with an Eksigent nano-flow liquid chromatograph (LC) (SCIEX, Framingham, MA, USA) and a Q-Exactive plus Orbitrap connected with the Ultimate 3000 RSLC system (Thermo Fisher Scientific). The equipment was kept at the Mass Spectrometry Convergence Research Center in Kyungpook National University. For the LTQ Velos-Orbitrap, the peptide separation was processed by a homemade C12 reversed-phase column (12 cm × 75 μm inner diameter, packed with Jupiter C12 resin) (Phenomenes, Torrance, CA, USA). A linear gradient was set to 3–23% solvent B (99.9% acetonitrile with 0.1% formic acid) for 75 min with a flow rate of 300 nL/min. The ionized peptides used a nanospray ion source, and the top-10 data-dependent modes were set. The range of the full MS is from 150 to 2000 *m*/*z*. For the Q-Exactive plus Orbitrap, the peptides were separated at the Easy-Spray column (15 cm × 75 μm inner diameter, PepMap 100 C18), serving at a flow rate of 300 nL/mL for 135 min. The range of full MS is from 350 to 2000 *m*/*z*.

### 4.7. Protein Identification and Quantification Using SILAC Labeling by LC–MS

For quantifying global proteins and Kac proteins, raw data were analyzed with MaxQuant 1.5 as the protein search engine for false discovery rate (FDR) analysis. The Uniprot human database (updated on Dec. 2018) was used for searching the data. The searching parameters were as follows: trypsin for enzyme digestion and two missed cleavages, 20 ppm of the mass tolerance of precursor, and 0.05 Da for tolerance production. Carbamidomethylation was specified as the fixed modification of the methionine oxidation, and lysine acetylation (+42.0106 Da) was chosen as the variable modification. FDR was set at <1% for protein identification. The peptides confidence was set as high for peptides filter. Unique protein peptides were selected for relative protein quantification. The normalization to Kac quantification was calculated as its protein ratios. The MS proteomics data were deposited in the ProteomeXchange Consortium via the PRIDE partner repository using the dataset identifier PXD 027496 [46].

### 4.8. Pyruvate Kinase Activity Assay

The pyruvate kinase (PK) activity assay kit (Abcam, Cambridge, UK, Ab83432) was used to measure pyruvate kinase activity between the control and the sepsis groups. All procedures were followed according to the manufacturer’s manual. In brief, 1 × 10^6^ cell pellets were prepared to extract with four volumes of assay buffer and bring the volume to 50 μL/well with PK assay buffer of each sample. The standard linear curve was generated by a serial dilution of 1 nmol/μL of a standard pyruvate solution. It was calculated using the amount of enzyme that transferred a phosphate group from phosphoenolpyruvate (PEP) to ADP, yielding 1.0 μmol of pyruvate per minute at 25 °C. All reacted standards and samples were subjected to colorimetric measurement at 570 nm of a microplate reader.

### 4.9. Sample Preparation for Lactate Quantification

A standard lactate mixture was prepared with 10 μg/μL in water and then 10 μL of the mixture were transferred into a new tube and dried. Next, 100 μL of water was added and derivatized. Cell pellets of each group were obtained after removal of media and washed PBS at 1 × 10^6^ cells. Next, 100 μL of 80% MeOH was added and mixed by vortex and then incubated on ice for 15 min. After centrifugation at 13,000× *g* for 3 min at 4. The sample extracts were dried and redisssolved with 100 μL of water. Next, the derivatization process was started by adding 50 μL of 1 M *O*-BHA and 50 μL of 1 M EDC. Two reagents—*O*-BHA and EDC—were prepared in pyridine buffer (pH 5). The samples were appropriately mixed and kept at room temperature for 1 h. Ethyl acetate (300 μL) was added to the samples after 1 h and was shaken (a strong vortex is not recommended) for 10 min using a multitube vortex. The upper organic layer was transferred to new tubes. Addition of ethyl acetate and separation of the organic layer was repeated at least thrice. All the organic phase obtained from the repeated addition of ethyl acetate was combined and dried using speed vacuum. The samples were reconstituted with 1 mL of 50% methanol containing 0.5 μm reserpine as internal standard and centrifuged at 13,000× *g* for 10 min at 4 °C. The supernatant was transferred to the vials, and 5 μL was injected for LC–MS/MS analysis.

### 4.10. Determination of Lactate Level by LC–MS/MS

An HR-MS coupled with ultrahigh-performance liquid chromatography (UHPLC) detected the derivatized lactate and pyruvate. The UHPLC system Dionex Ultimate 3000 (Dionex Softron GmbH, Germering, Germany) was used. A reverse-phase liquid chromatography column, the 150 mm × 3 mm, 3-μm Shim-pack CIS C18 column (Shimadzu Corporation, Kyoto, Japan), at 40 °C temperature was used to separate the derivatized lactate and pyruvate. The gradient elution state for separation was as follows: 5% of B between 0 and 2 min; 5–65% of B between 2 and 10 min; 65–90% of B between 10 and 11 min; 90% of B between 11 and 13 min; 90–95% of B between 13 and 14 min; and 5% of B between 14 and 17 min. The MS detection was performed on Q-Exactive Focus quadrupole-Orbitrap MS (Thermo Fisher Scientific, Bremen, Germany) equipped with a heated electrospray ionization (HESI-II) ion probe. The lactate and pyruvate were detected in positive ion mode after derivatization. Moreover, MS-grade solvents were used as the mobile phase with a flow rate of 400 L/min in the gradient elution mode. Water with 0.1% formic acid and 0.1% formic acid in ACN were solvent A and solvent B, respectively, for the LC–MS analysis.

### 4.11. Bioinformatics

The DAVID software was utilized for the GO annotation and the KEGG pathway (https://david.ncifcrf.gov/, accessed on 17 June 2021). The GO enrichment analysis showed that six identified clusters were related to specific biological processes, molecular functions, and cellular components. The Perseus program was used to clarify the volcano plot and heat map between groups.

### 4.12. Statistical Analysis

Data are presented as mean ± SE and were analyzed using a *t*-test and a one-way ANOVA, followed by multiple comparisons. *p* < 0.05 (*) and *p* < 0.01 (**) were considered statistically significant.

## Figures and Tables

**Table 1:** The list of upregulated Kac proteins in LPS-induced SILD in HepG2 cells for 24 h.

No.	Protein Name	Proteins	Gene Name	Modified Sequence	Score	Positions	Charge	Ratio *
1	Calreticulin	P27797	*CALR*	_GK(ac)NVLINK_	76.478	K153	2	5.57
2	Myosin-9	P35579	*MYH9*	_K(ac)FDQLLAEEK_	63.473	K1445	2	3.20
3	Nucleoside diphosphate kinase	Q32Q12	*NME1-NME2*	_FEQK(ac)GFR_	100.19	K56	2	3.05
4	CCAAT/enhancer-binding protein gamma	P53567	*CEBPG*	_AVAPSK(ac)QSK_	104.89	K50	2	2.75
5	Aldo-keto reductase family 1 member C3	P42330	*AKR1C3*	_SK(ac)IADGSVK_	72.2	K68	2	2.63
6	Heterogeneous nuclear ribonucleoprotein U	A0A1 × 7SBS1	*HNRNPU*	_K(ac)AEVEGKDLPEHAVLK_	68.751	K539	3	2.57
7	60S ribosomal protein L35	P42766	*RPL35*	_LNK(ac)HEENLK_	75.652	K97	3	2.48
8	60S ribosomal protein L3	P39023	*RPL3*	_FIDTTSK(ac)FGHGR_	41.242	K373	3	2.30
9	Protein disulfide-isomerase	P07237	*P4HB*	_TAAESFK(ac)GK_	76.228	K283	2	2.18
10	40S ribosomal protein SA	C9J9K3	*RPSA*	_AVLK(ac)FAAATGATPIAGR_	118.66	K89	2	2.08
11	Peroxiredoxin-2	P32119	*PRDX2*	_ATAVVDGAFK(ac)EVK_	119.88	K26	2	2.06
12	Alpha-enolase	P06733	*ENO1*	_IDK(ac)LM(ox)IEM(ox)DGTENK_	171.15	K92	2	2.04
13	Fructose-bisphosphate aldolase A	P04075	*ALDOA*	_AAQEEYVK(ac)R_	113.62	K330	2	2.04
14	Heat shock protein HSP 90-alpha	P07900	*HSP90AA1*	_FYEQFSK(ac)NIK_	66.056	K443	2	2.03
15	Pyruvate kinase PKM	P14618	*PKM*	_EM(ox)IK(ac)SGM(ox)NVAR_	43.68	K66	2	2.00

*, Kac site ratio (24 h vs. 0 h) normalized by their protein ratio (24 h vs. 0 h). The Kac site ratio in the 24 h group (“heavy”) was divided by the 0 h group (“light”) same as calculated for its protein ratio. Then, the Kac ratio (24 h vs. 0 h) for its protein, peptide, and site was normalized by protein ratio (24 h vs. 0 h).

## Data Availability

Data available in a publicly accessible repository.

## Supplementary Materials

The following are available online at https://www.mdpi.com/article/10.3390/ijms22168529/s1.

## References

[1] Liu T.F., Brown C.M., El Gazzar M., McPhail L., Millet P., Rao A., Vachharajani V.T., Yoza B.K., McCall C.E. (2012). Fueling the flame: Bioenergy couples metabolism and inflammation. J. Leukoc Biol..

[2] Calandra T., Echtenacher B., Roy D.L., Pugin J., Metz C.N., Hültner L., Heumann D., Männel D., Bucala R., Glauser M.P. (2000). Protection from septic shock by neutralization of macrophage migration inhibitory factor. Nat. Med..

[3] Baranova I.N., Souza A.C., Bocharov A.V., Vishnyakova T.G., Hu X., Vaisman B.L., Amar M.J., Chen Z., Kost Y., Remaley A.T. (2016). Human SR-BI and SR-BII potentiate lipopolysaccharide-induced inflammation and acute liver and kidney injury in mice. J. Immunol..

[4] Arumanayagam S., Arunmani M. (2015). Hepatoprotective and antibacterial activity of Lippia nodiflora Linn. against lipopolysaccharides on HepG2 cells. Pharmacogn. Mag..

[5] Dhainaut J.F., Marin N., Mignon A., Vinsonneau C., Sprung C. (2001). Hepatic response to sepsis: Interaction between coagulation and inflammatory processes. Crit. Care Med..

[6] Kramer L., Jordan B., Druml W., Bauer P., Metnitz P.G.H. (2007). Incidence and prognosis of early hepatic dysfunction in critically ill patients—A prospective multicenter study. Crit. Care Med..

[7] Yan J., Li S., Li S. (2014). The role of the liver in sepsis. Int. Rev. Immunol..

[8] Becker K.L., Snider R., Nylen E.S. (2010). Procalcitonin in sepsis and systemic inflammation: A harmful biomarker and a therapeutic target. Br. J. Pharmacol..

[9] Audagnotto M., Peraro M.D. (2017). Protein post-translational modifications: In silico prediction tools and molecular modeling. CSBJ.

[10] Sabari B.R., Zhang D., Allis C.D., Zhao Y. (2017). Metabolic regulation of gene expression through histone acylations. Nat. Rev. Mol. Cell Biol..

[11] Strahl B.D., Allis C.D. (2000). The language of covalent histone modifications. Nature.

[12] Zhao S., Xu W., Jiang W., Yu W., Lin Y., Zhang T., Yao J., Zhou L., Zeng Y., Li H. (2010). Regulation of cellular metabolism by protein lysine acetylation. Science.

[13] Christensen D.G., Xie X., Basisty N., Byrnes J., McSweeney S., Schilling B., Wolfe A.J. (2019). Post-translational protein acetylation: An elegant mechanism for bacteria to dynamically regulate metabolic functions. Front. Microbiol..

[14] El Ramy R., Magroun N., Messadecq N., Gauthier L.R., Boussin F.D., Kolthur-Seetharam U., Schreiber V., McBurney M.W., Sassone-Corsi P., Dantzer F. (2009). Functional interplay between Parp-1 and SirT1 in genome integrity and chromatin-based processes. Cell. Mol. Life Sci..

[15] Herskovits A.Z., Guarente L. (2013). Sirtuin deacetylases in neurodegenerative diseases of aging. Cell Res..

[16] Wang X., Buechler N.L., Woodruff A.G., Long D.L., Zabalawi M., Yoza B.K., McCall C.E., Vachharajani V. (2018). Sirtuins and immuno-metabolism of sepsis. Int. J. Mol. Sci.

[17] Seto E., Yoshida M. (2014). Erasers of histone acetylation: The histone deacetylase enzymes. Cold Spring Harb. Perspect. Biol..

[18] Tao Y., Huang C., Huang Y., Hong L., Wang H., Zhou Z., Qiu Y. (2015). SIRT4 suppresses inflammatory responses in human umbilical vein endothelial cells. Cardiovasc. Toxicol..

[19] Du J., Zhou Y., Su X., Yu J.J., Khan S., Jiang H., Kim J., Woo J., Kim J.H., Choi B.H. (2011). Sirt5 is a NAD-dependent protein lysine demalonylase and desuccinylase. Science.

[20] Zhang Y., Anoopkumar-Dukie S., Mallik S.B., Davey A.K. (2021). SIRT1 and SIRT2 modulators reduce LPS-induced inflammation in HAPI microglial cells and protect SH-SY5Y neuronal cells in vitro. J. Neural Transm..

[21] Li F.L., Liu J.P., Bao R.X., Yan G., Feng X., Xu Y.P., Sun Y.P., Yan W., Ling Z.Q., Xiong Y. (2018). Acetylation accumulates PFKFB3 in cytoplasm to promote glycolysis and protects cells from cisplatin-induced apoptosis. Nat. Commun..

[22] Miyai M., Kanayama T., Hyodo F., Kinoshita T., Ishihara T., Okada H., Suzuki H., Takashima S., Wu Z., Hatano Y. (2021). Glucose transporter Glut1 controls diffuse invasion phenotype with perineuronal satellitosis in diffuse glioma microenvironment. Neurooncol. Adv..

[23] Li L., Chen Z., Fu W., Cai S., Zeng Z. (2018). Emerging evidence concerning the role of sirtuins in sepsis. Crit. Care Res. Pract..

[24] Vachharajani V.T., Liu T., Wang X., Hoth J.J., Yoza B.K., McCall C.E. (2016). Sirtuins link inflammation and metabolism. J. Immunol. Res..

[25] Hasegawa A., Iwasaka H., Hagiwara S., Asai N., Nishida T., Noguchi T. (2012). Alternate day calorie restriction improves systemic inflammation in a mouse model of sepsis induced by cecal ligation and puncture. J. Surg. Res..

[26] Xu W., Lu Y., Yao J., Li Z., Chen Z., Wang G., Jing H., Zhang X., Li M., Peng J. (2014). Novel role of resveratrol: Suppression of high-mobility group protein box 1 nucleocytoplasmic translocation by the upregulation of sirtuin 1 in sepsis-induced liver injury. Shock.

[27] Hwang J.S., Choi H.S., Ham S.A., Yoo T., Lee W.J., Paek K.S., Seo H.G. (2015). Deacetylation-mediated interaction of SIRT1-HMGB1 improves survival in a mouse model of endotoxemia. Sci. Rep..

[28] Liu T.F., Yoza B.K., El Gazzar M., Vachharajani V.T., McCall C.E. (2011). NAD^+^-dependent SIRT1 deacetylase participates in epigenetic reprogramming during endotoxin tolerance. J. Biol. Chem..

[29] Vachharajani V.T., Liu T., Brown C.M., Wang X., Buechler N.L., Wells J.D., Yoza B.K., McCall C.E. (2014). SIRT1 inhibition during the hypoinflammatory phenotype of sepsis enhances immunity and improves outcome. J. Leukoc. Biol..

[30] Fernandes C.A., Fievez L., Neyrinck A.M., Delzenne N.M., Bureau F., Vanbever R. (2012). Sirtuin inhibition attenuates the production of inflammatory cytokines in lipopolysaccharide-stimulated macrophages. Biochem. Biophys. Res. Commun..

[31] Mahlknecht U., Voelter-Mahlknecht S. (2009). Fluorescence in situ hybridization and chromosomal organization of the sirtuin 4 gene (Sirt4) in the mouse. Biochem. Biophys. Res. Commun..

[32] Yang L., Ma X., He Y., Yuan C., Chen Q., Li G., Chen X. (2017). Sirtuin 5: A review of structure, known inhibitors and clues for developing new inhibitors. Sci. China Life Sci..

[33] Lee S.G., Song J., Park D.W., Moon S., Cho H.J., Kim J.Y., Park J., Cha J.H. (2021). Prognostic value of lactate levels and lactate clearance in sepsis and septic shock with initial hyperlactatemia: A retrospective cohort study according to the Sepsis-3 definitions. Medicine.

[34] Rivas A.M., Nugent K. (2021). Hyperglycemia, insulin, and insulin resistance in sepsis. Am. J. Med. Sci..

[35] Cao C., Gao T., Cheng Y., Cheng M., Su T., Xi F., Wu C., Yu W. (2018). Hypothalamic AMPK-induced autophagy ameliorates hypercatabolism in septic rats by regulating POMC expression. Biochem. Biophys. Res. Commun..

[36] Lee J.H., Kim S.W., Kim J.H., Kim H.J., Um J., Jung D.W., Williams D.R. (2021). Lithium chloride protects against sepsis-induced skeletal muscle atrophy and cancer cachexia. Cells.

[37] Rello J., Valenzuela-Sánchez F., Ruiz-Rodriguez M., Moyano S. (2017). Sepsis: A review of advances in management. Adv. Ther..

[38] Andersen L.W., Mackenhauer J., Roberts J.C., Berg K.M., Cocchi M.N., Donnino M.W. (2013). Etiology and therapeutic approach to elevated lactate levels. Mayo Clin. Proc..

[39] Levy B., Desebbe O., Montemont C., Gibot S. (2008). Increased aerobic glycolysis through β2 stimulation is a common mechanism involved in lactate formation during shock states. Shock.

[40] Zahra K., Dey T., Mishra S.P., Pandey U. (2020). Pyruvate Kinase M2 and Cancer: The role of PKM2 in promoting tumorigenesis. Front. Oncol..

[41] Yang L., Xie M., Yang M., Yu Y., Zhu S., Hou W., Kang R., Lotze M.T., Billiar T.R., Wang H. (2014). PKM2 regulates the Warburg effect and promotes HMGB1 release in sepsis. Nat. Commun..

[42] Garcia-Alvarez M., Marik P., Bellomo R. (2014). Sepsis-associated hyperlactatemia. Crit. Care.

[43] Zhang Z.H., Zhang H., Wang Y.R., Liu X.L., Huang H., Xu X.H. (2019). SIRT 1 binding with PKM and NSE and modulate their acetylation and activities. Biochim. Biophys. Acta Proteins Proteom..

[44] Xiong Y., Lei Q.Y., Zhao S., Guan K.L. (2011). Regulation of glycolysis and gluconeogenesis by acetylation of PKM and PEPCK. Cold Spring Harb. Symp. Quant. Biol..

[45] Wang F., Wang K., Xu W., Zhao S., Ye D., Wang Y., Xu Y., Zhou L., Chu Y., Zhang C. (2017). SIRT5 desuccinylates and activates pyruvate kinase M2 to block macrophage IL-1β production and to prevent DSS-induced colitis in mice. Cell Rep..

[46] Vizcaíno J.A., Deutsch E.W., Wang R., Csordas A., Reisinger F., Ríos D., Dianes J.A., Sun Z., Farrah T., Bandeira N. (2014). ProteomeXchange provides globally coordinated proteomics data submission and dissemination. Nat. Biotechnol..

