# Supplementary material for: Global Lysine Acetylome Analysis of LPS-Stimulated HepG2 Cells Identified Hyperacetylation of PKM2 as a Metabolic Regulator in Sepsis
Source: Int J Mol Sci. 2021 Aug 8;22(16):8529. doi: 10.3390/ijms22168529 (PMC8395202; doi:10.3390/ijms22168529)
Supplement: Supplementary file 1 [file ijms-22-08529-s001.zip › ijms-1283398 suppl/Supplemental inform_proof revision.pdf]

Supplemental information

Global lysine acetylome analysis of LPS-stimulated HepG2 cells identified hyperacetylation of PKM2 as a metabolic regulator in sepsis

Ann-Yae Na[1], Sanjita Paudel[1], Soyoung Choi[1], Jun Hyung Lee[2], Min-Sik Kim[2], Jong-Sup Bae[1], Sangkyu Lee[1,*]

[1]BK21 FOUR Community-Based Intelligent Novel Drug Discovery Education Unit, College of Pharmacy and Research Institute of Pharmaceutical Sciences, Kyungpook National University, Daegu 41566, Republic of Korea

[2]Department of New Biology, DGIST, Daegu, 42988, Republic of Korea

[*]**Corresponding Author**: Sangkyu Lee, Ph.D., Associate Professor, College of Pharmacy, Kyungpook National University, Daegu 41566, Republic of Korea, E-mail, sangkyu@knu.ac.kr; Phone, +82 53 950 8571; Fax, +82 53 950 8557

Legend of Tables

Table S1. List of all the identified proteins

Table S2. Protein list in Clusters 1-5

Table S3. All identified list of acetylated peptides

Table S4. The primer information of SIRT 1-7

Table S5. The antibody information of SIRT 1-7

Legend of Figures

Figure S1. The level of inflammatory factors (A) and SIRT 1-7 (B) determined using Western blotting. HepG2 cells were incubated with LPS (1 μg/mL) for different durations.

Figure S2. Overview of global proteomics analysis from sepsis-induced liver dysfunction (SILD) *in vitro* (A) The number of identified protein and quantified proteins and DEPs of group (B) Venn diagram of protein quantification in triplicate analyzing results of the LPS 6 h (left) and the 24 h (right) groups compared with that of the control group.

Figure S3. Correlation analysis of experimental results with report ion ratios among biological triplicate samples in sepsis-induced liver dysfunction (SILD) *in vitro*.

Figure S4. Protein expression dynamic clusters based on global protein expression patterns at each stage of sepsis-induced liver dysfunction (SILD) *in vitro*. The functional GO and KEGG were shown at each cluster.

Figure S5. Characterization of lysine acetylation (Kac) in SILD *in vitro*. (A) Distribution of lysine acetylation (Kac) sites per protein. (B) Correlation of biological duplicate by intensity ratio in Kac. (C) Functional enrichment of gene ontology (GO).

Figure S6. The proteins interaction network with a strong interaction (highest confidence 0.09) among the associated in glycolysis pathway.

Figure S7. Validation of comparative proteome analysis using Western blotting. Western blot analysis for PKM2 protein ex-pression (A). The PKM and PKM acetylation levels were determined using Western blotting after immunoprecipitation using an anti-PKM antibody (B).

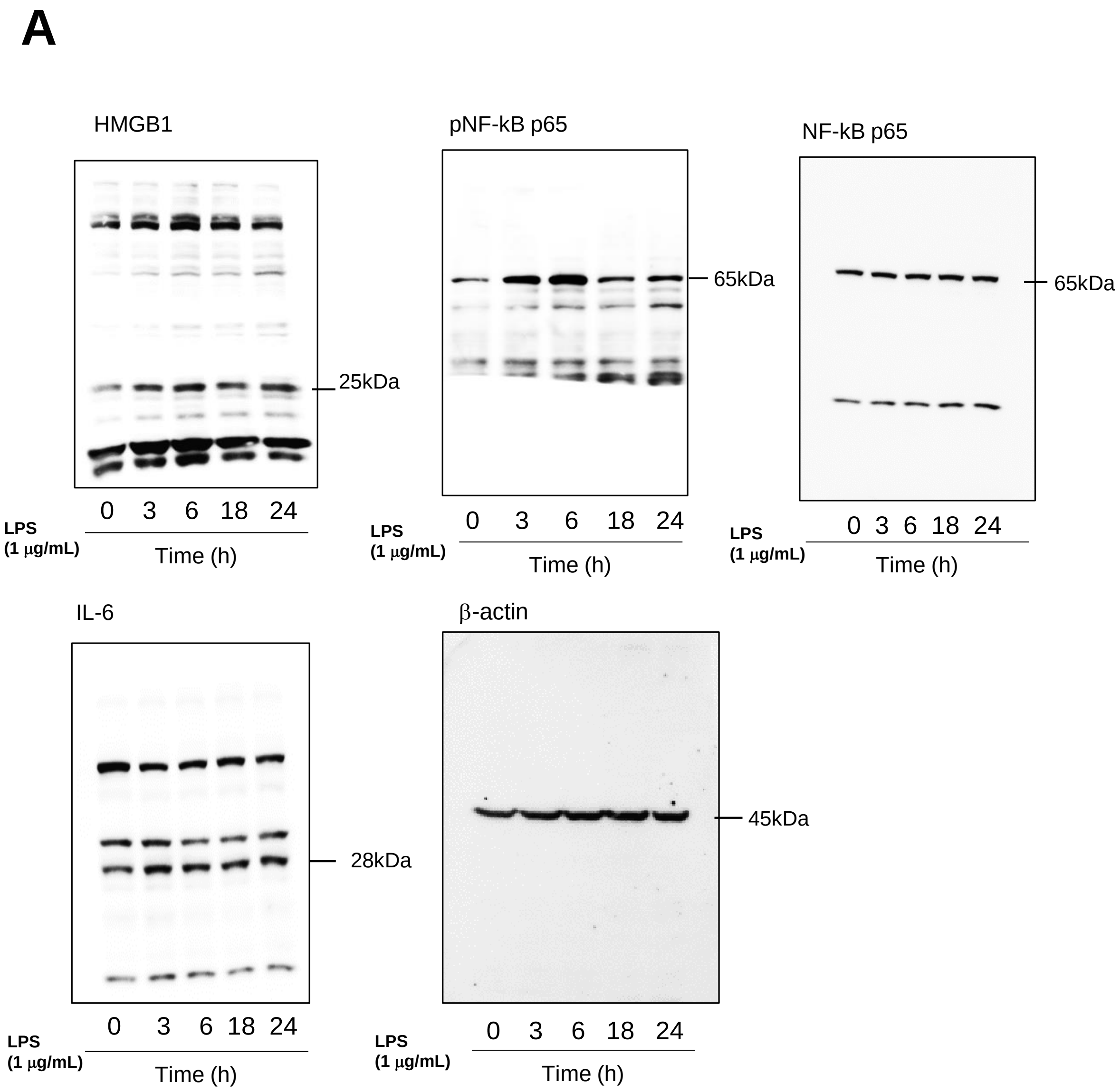

Figure S1. The level of inflammatory factors (A) and SIRT 1-7 (B) determined using Western blotting. HepG2 cells were incubated with LPS (1 μg/mL) for different durations (continued).

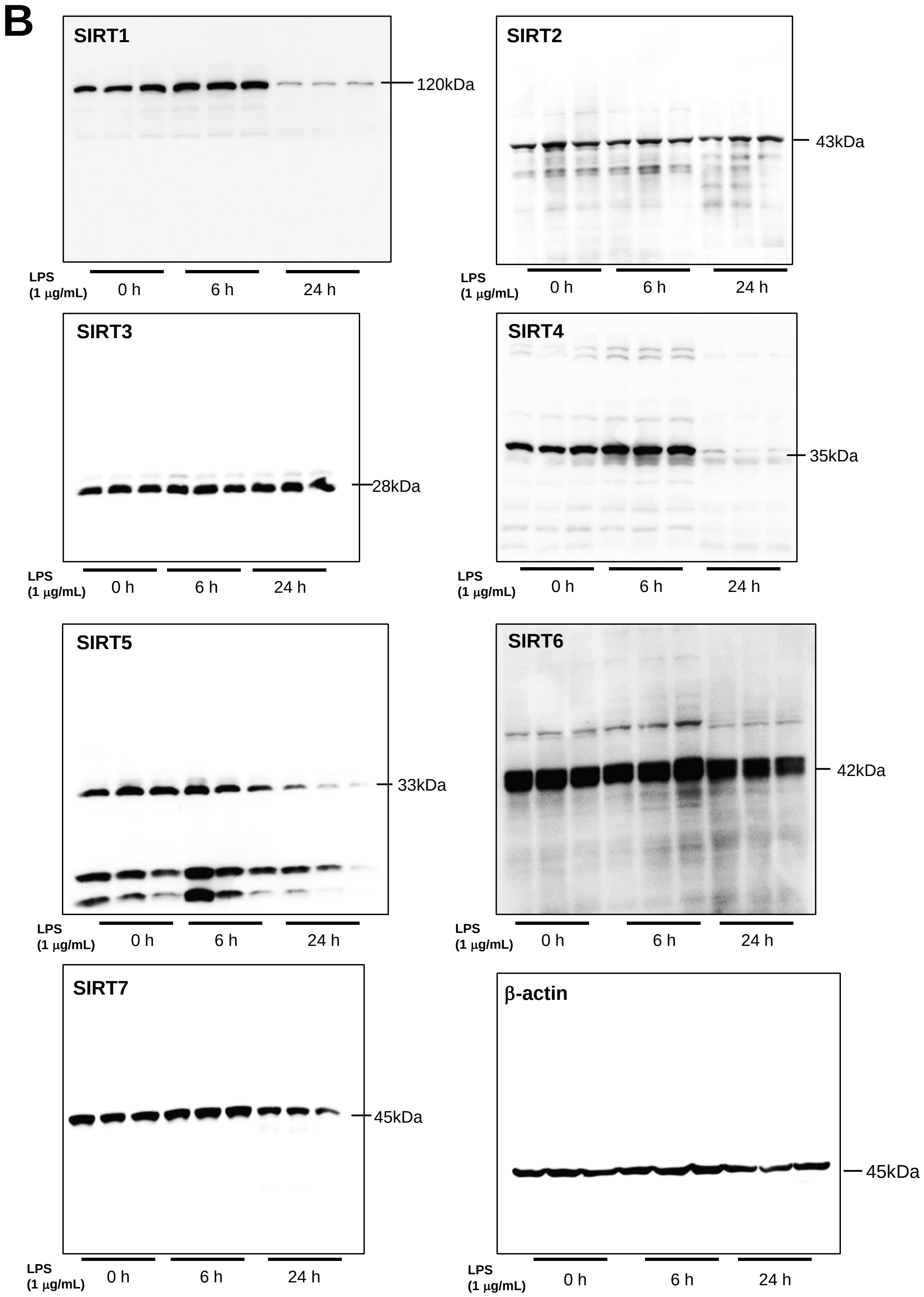

Figure S1. The level of inflammatory factors (A) and SIRT 1-7 (B) determined using Western blotting. HepG2 cells were incubated with LPS (1 μg/mL) for different durations (continued).

A

	LPS 6 h	LPS 24 h
# Ident.	3,167	
# Quant.	2,256	

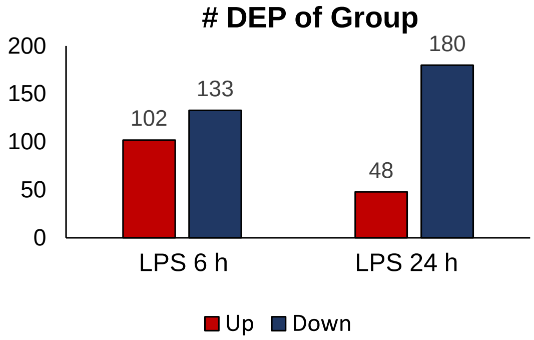

B

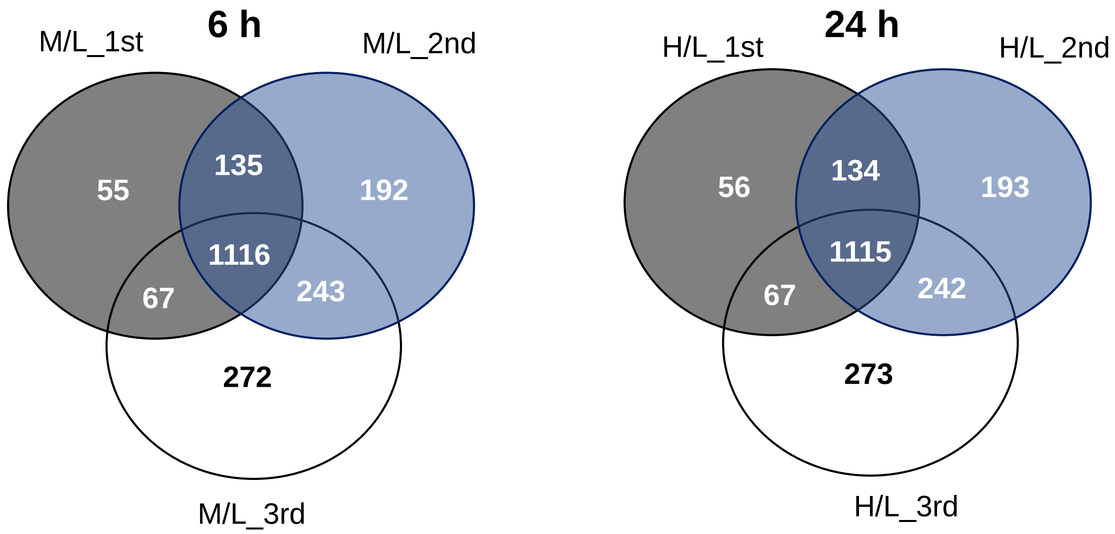

Figure S2. Overview of global proteomics analysis from sepsis-induced liver dysfunction (SILD) *in vitro* (A) The number of identified protein and quantified proteins and DEPs of group (B) Venn diagram of protein quantification in triplicate analyzing results of the LPS 6 h (left) and the 24 h (right) groups compared with that of the control group.

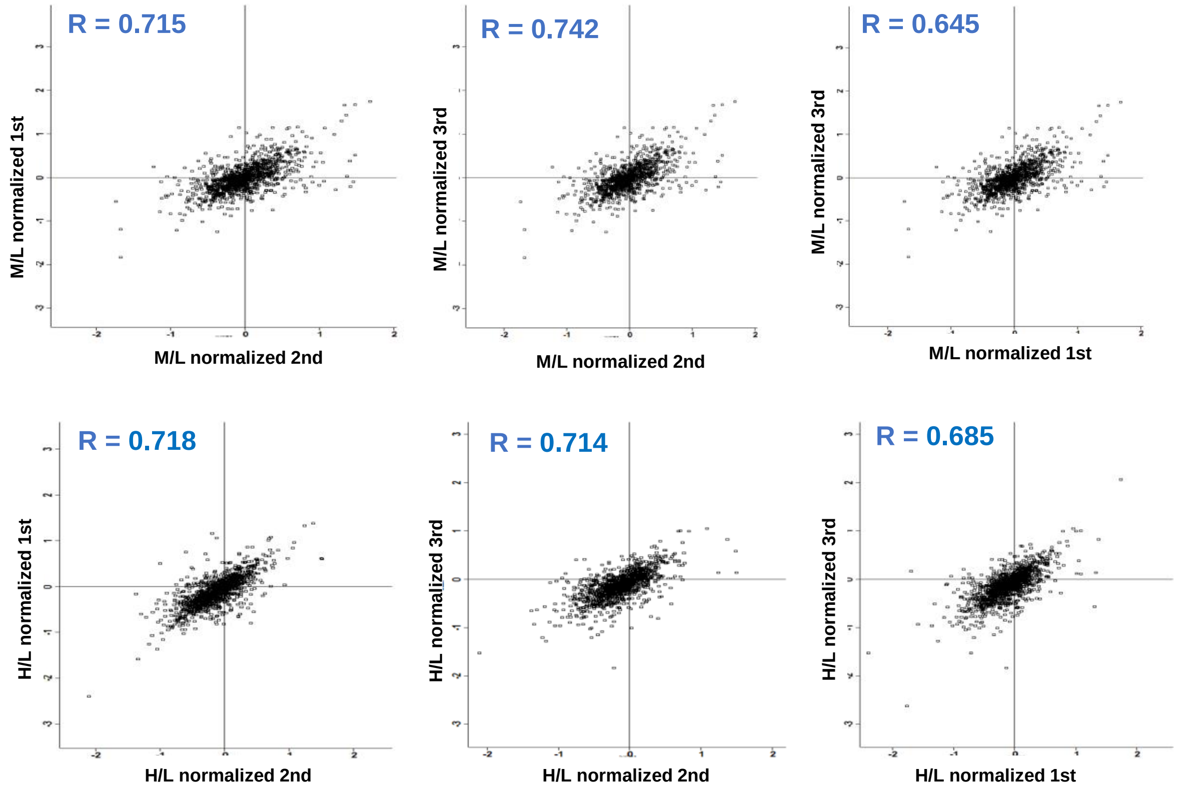

Figure S3. Correlation analysis of experimental results with report ion ratios among biological triplicate samples in sepsis-induced liver dysfunction (SILD) *in vitro*.

Cluster 2

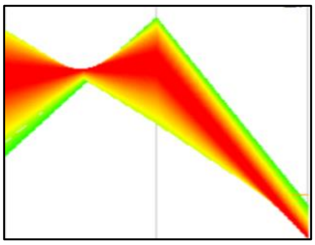

	Terms	*p*-value
GOBP	mRNA splicing, via spliceosome	29.68
GOBP	RNA splicing	11.02
GOCC	extracellular exosome	35.21
GOCC	mitochondrion	28.92
GOMF	poly(A) RNA binding	74.00
GOMF	RNA binding	14.47
KEGG	Carbon metabolism	18.48
KEGG	Spliceosome	14.21

Cluster 3

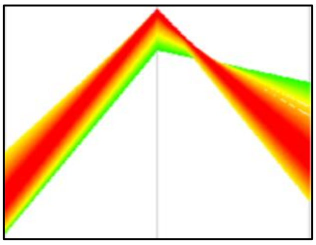

	Terms	*p-value*
GOBP	SRP-dependent cotranslational protein targeting to membrane	37.24
GOBP	translational initiation	36.60
GOCC	extracellular exosome	50.36
GOCC	cytosol	48.85
GOMF	poly(A) RNA binding	51.57
GOMF	structural constituent of ribosome	24.07
KEGG	Ribosome	18.92
KEGG	Proteasome	11.85

Cluster 4

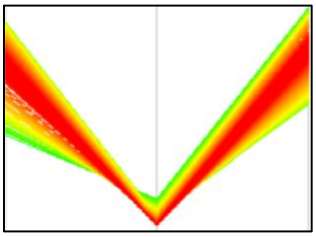

	Terms	*p*-value
GOBP	cell-cell adhesion	10.58
GOBP	translation	6.60
GOCC	extracellular exosome	22.72
GOCC	cytosol	17.95
GOMF	poly(A) RNA binding	16.79
GOMF	cadherin binding involved in cell-cell adhesion	13.26
KEGG	Ribosome	4.55
KEGG	Protein processing in ER	3.63

Cluster 5

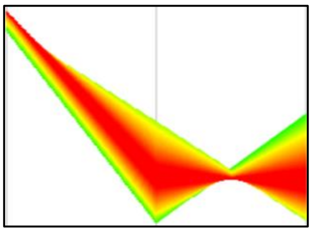

	Terms	*p*-value
GOBP	cell-cell adhesion	17.49
GOBP	mRNA splicing, via spliceosome	17.00
GOCC	mitochondrion	34.68
GOCC	nucleoplasm	34.26
GOMF	poly(A) RNA binding	68.82
GOMF	cadherin binding involved in cell-cell adhesion	18.54
KEGG	Spliceosome	14.80
KEGG	Metabolic pathways	10.08

Figure S4. Protein expression dynamic clusters based on global protein expression patterns at each stage of sepsis-induced liver dysfunction (SILD) *in vitro*. The functional GO and KEGG were shown at each cluster.

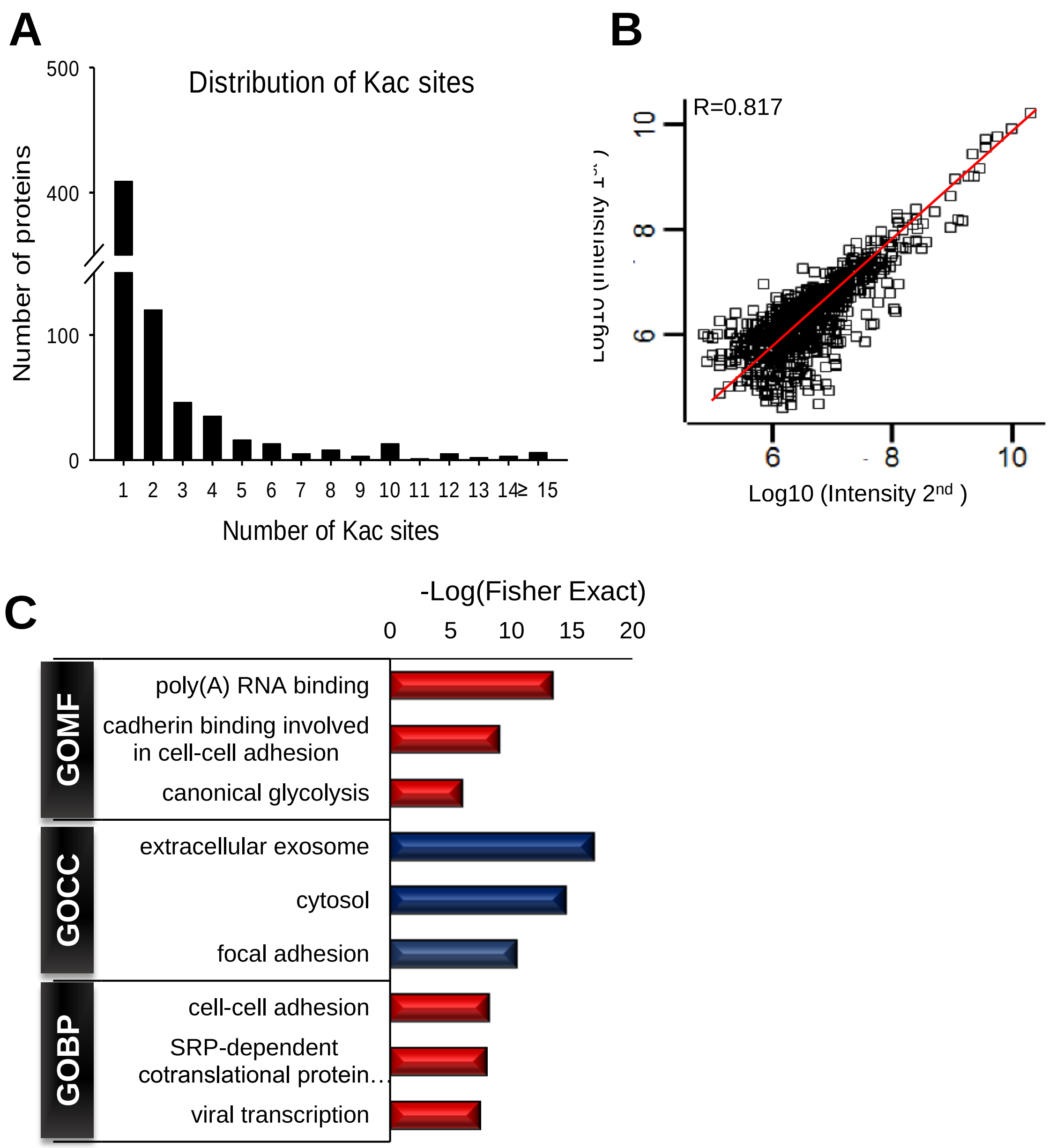

Figure S5. Characterization of lysine acetylation (Kac) in SILD *in vitro*. (A) Distribution of lysine acetylation (Kac) sites per protein. (B) Correlation of biological duplicate by intensity ratio in Kac. (C) Functional enrichment of gene ontology (GO).

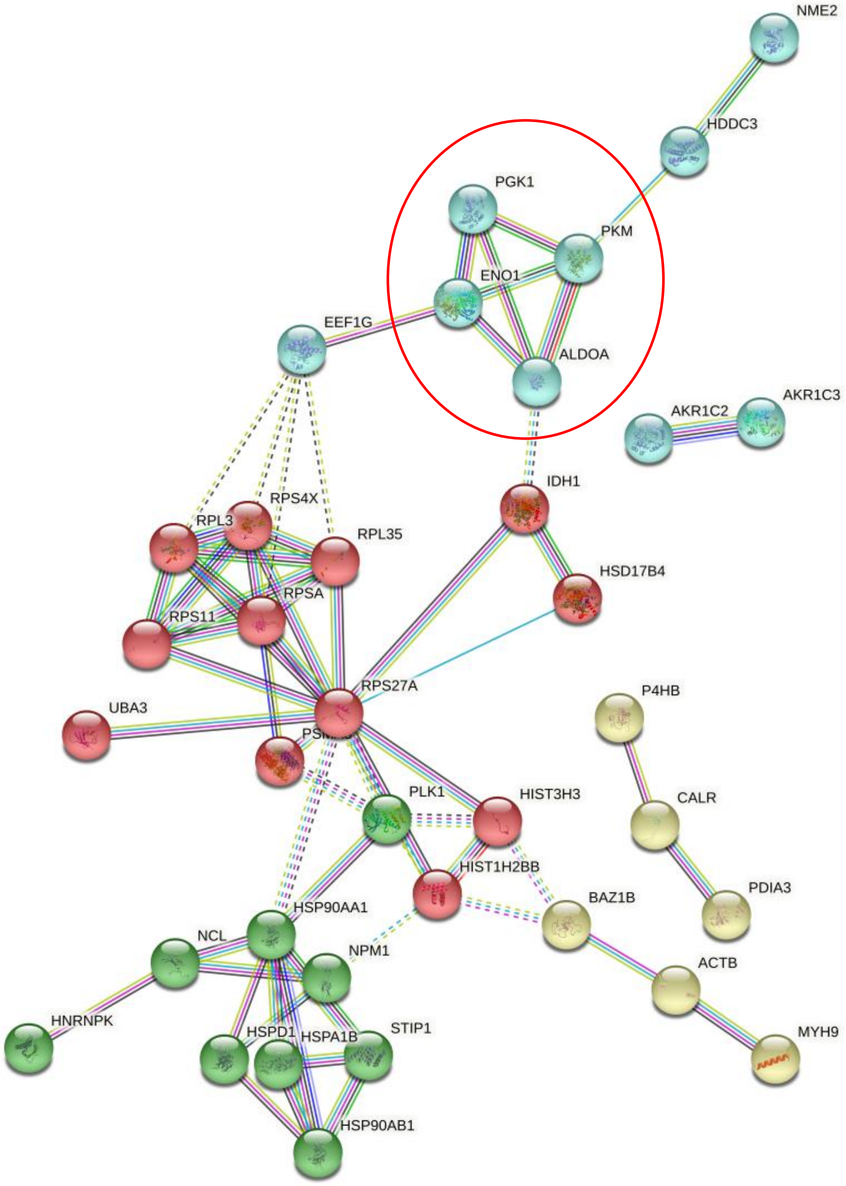

Figure S6. The proteins interaction network with a strong interaction (highest confidence 0.09) among the associated in glycolysis pathway.

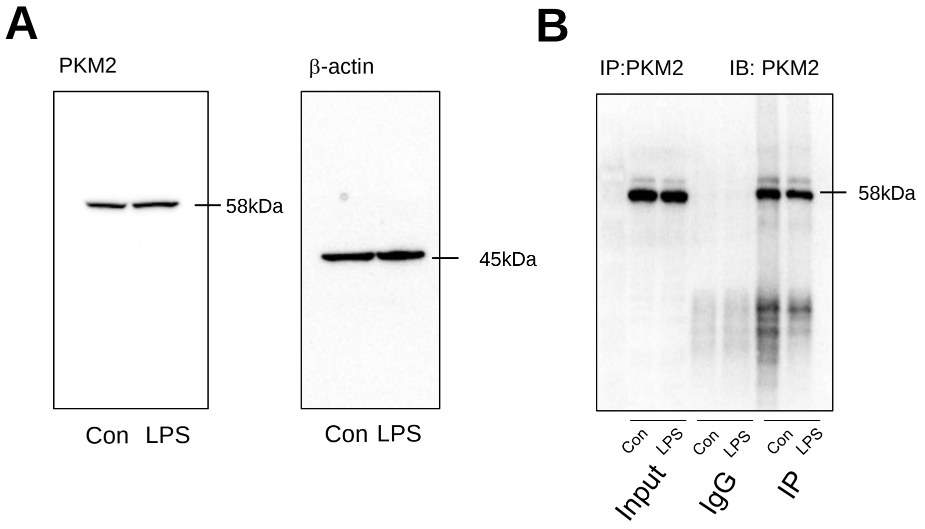

Figure S7. Validation of comparative proteome analysis using Western blotting. Western blot analysis for PKM2 protein ex-pression (A). The PKM and PKM acetylation levels were determined using Western blotting after immunoprecipitation using an anti-PKM antibody (B).

Table S4. The primer information of SIRT 1-7

Gene	Forward	Reverse	Accession No.
SIRT1	GAGCTGGGGTGTCTGTTTCA	GGAAGTCTACAGCAAGGCGA	NM_012238.5
SIRT2	CCTCCTCCTGGTCATGGGTA	CGAGGCCCATAATCATCCCC	NM_012237.4
SIRT3	CCCAGTGGCATTCCAGACTT	AGGGCTTGGGGTTGTGAAA	NM_012239.6
SIRT4	CTCGAAAGCCTCCATTGGGT	CTGGTATCCCCGATTCGGTG	NM_012240.2
SIRT5	TCAGGGGTTCACAGAATGG	ACCTTACCCCTACCCCATCC	NM_012241.5
SIRT6	GCAGTCTTCCAGTGTGGTGT	GCTCTCAAAGGTGGTGTCGA	NM_016539.4
SIRT7	CAGGTGGCAGGATCCCATTT	CCTCCTCTCTGCTTCTGCAC	NM_016538.3
β-actin	AATCTGGCACCACACCTTCTAC	ATAGCACAGCCTGGATAGCA AC	NM_001101.5

Table S5. The antibody information of SIRT 1-7

Protein	Cat. No.	Protein	Cat. No.	Protein	Cat. No.
SIRT1	CST #3931	PKM2	Proteintech #15822-1-AO	Kglu	PTM-1151
SIRT2	CST #12672	HMGB1	Abcam 79823	Kbut	PTM-301
SIRT3	CST #5490	IL-6	CST #12153	Klac	PTM-1401
SIRT4	Abcam 231137	NFκB P65	CST #4764	Kben	PTM-762
SIRT5	Abcam 154082	pNFκB p65	CST #3303	Kcro	PTM-502
SIRT6	CST #12486S	Kac	PTM-101	Kpro	PTM-201
SIRT7	CST #5360S	Ksu	PTM-401		
β-actin	CST #4967	Kmal	PTM-901		
